# COVID-19, Macroeconomic and Sustainability Shocks, Moral Hazard and Resolution of Systemic Banking Crises: Designing Appropriate Systems of Public Support

**DOI:** 10.1007/s40804-022-00255-1

**Published:** 2022-08-09

**Authors:** Douglas W. Arner, Emilios Avgouleas, Evan C. Gibson

**Affiliations:** 1 Kerry Holdings Professor in Law, Hong Kong Research Grants Council Senior Research Fellow in Digital Finance and Sustainable Development and Associate Director, HKU-Standard Chartered Foundation FinTech Academy, University of Hong Kong, Hong Kong, China; 2 Senior Fellow, Melbourne Law School, University of Melbourne, Melbourne, Australia; 3 Chair in International Banking Law and Finance, University of Edinburgh, Edinburgh, UK; 4 Visiting Professor, School of European Political Economy, Luiss Guido Carli, Rome, Italy; 5 Research Assistant Professor, HKU-Standard Chartered Foundation FinTech Academy, Faculty of Law, University of Hong Kong, Hong Kong, China

**Keywords:** Banking crisis, Bailouts, Bank regulation, Non-performing loans, Asset management companies, COVID-19

## Abstract

Banks have so far weathered well the financial turbulence caused by COVID-19 while at the same time being central in the economic and financial response. As the crisis moves from its initial phase as a short-term liquidity shock, the financial sector is facing increasing volumes of non-performing loans, raising the spectre of a banking solvency crisis. In economies already burdened with low-quality assets, the COVID-19 fallout is intensifying existing problems with legacy loans heightening the risk of a banking crisis. These issues are now being worsened by the impact of inflation and the invasion of Ukraine. Thus, addressing increasing volumes of bad loans, while supporting the proper functioning of the financial system, is a major challenge with systemic repercussions for a range of economies. This paper identifies a great paradox: since the bank rescues of the 2008–9 Global Financial Crisis there has been a disproportionate focus on the liability side of bank balance sheets through resolution measures such as bail-in and the accumulation of bail-inable debt. Post-crisis bank resolution regimes have overlooked solutions lying within the asset side of bank balance sheets. This paper analyses historical evidence to argue that concentrating on a liability-focused approach to the exclusion of asset-side solutions is ill-conceived. An excessive accumulation of non-performing loans on the asset side of bank balance sheets inevitably renders resolution interventions on the liability/equity side ineffective or at the very least insufficient to maintain banking system viability and financial stability. Bank asset restructuring involving the use of asset management companies, asset protection schemes and even capital injections can play a critical role in achieving an expeditious restoration of banking systems’ health following a major macroeconomic, sustainability or financial crisis.

## Introduction

Banking crises are commonly a consequence of bank balance sheets overloaded with non-performing exposures (NPEs) and high leverage ratios.^1^ When an economy expands, credit standards are typically overly optimistic, supporting asset prices to increase above so-called fundamental values. Conversely, when the economic cycle contracts and default risk rises, banks tighten lending standards, increasing the cost and decreasing the supply of credit. In addition, by burdening capital, the debt overhang constrains credit and growth.^2^ Borrowers with high credit default risk are forced to de-lever by selling assets, which places downward pressure on asset prices.^3^ Asset price falls place pressure on bank balance sheets as collateral cover drops and borrowers face challenges in making repayments and accessing credit and liquidity, which is reflected by increases in non-performing loans (NPLs).^4^

As the economy enters recession, banks must manage balance sheet and liquidity stress, which in turn heightens solvency risk.^5^ When asset sales are used to shore up bank capital and liquidity buffers, and where the central bank does not sufficiently loosen interest rates or take other measures to boost the credit supply, such as asset purchases or so-called quantitative easing, fire sales and bank defaults are likely to follow, often the signal of the onset of a systemic financial crisis.^6^

To mitigate the impact of these risks, regulators—in normal times—should take preventative measures comprising: (1) prudential regulations including appropriate levels of loan pre-provisioning, loan-to-income and loan-to-value ratios, debt service coverage ratios, and micro and macroprudential capital, liquidity and leverage requirements; (2) close monitoring of NPL recognition, ratios and volumes; (3) disclosure and auditing requirements; (4) requirements for stress testing, resolvability assessments and contingency planning (such as in the context of recovery and resolution plans); (5) requirements for capital instruments which can be used in the context of restructuring (*bail-in*); and (6) design of appropriate systems for dealing with liquidity provision, resolution (including insolvency) and customer protection (e.g., deposit insurance). Ideally, these measures would be countercyclical, allowing banks to maintain lending in a downturn through the release of capital buffers, and, at the same time, able to manage any bank failures.^7^ Following major financial regulatory reforms in the wake of the 2008–9 Global Financial Crisis (GFC), this has generally been the approach during 2020–22 in the context of the COVID-19 crisis. The effectiveness of such measures depends to a great extent on the magnitude of a crisis, its origins and the overall levels of bad assets.

In orderly market conditions, the measure of bank losses from NPLs is reflected on the balance sheet—typically the difference between an asset’s *net present value*^8^ plus bank provisions (so-called *book value*) and the ultimate amount recovered. The recovery amount is contingent on the ability of the borrower to restructure the debt contract or on the ease with which the creditor can dispose of collateral in a fairly valued market. Loss-given default is minimised where the legal system is functioning in a pro-creditor environment (including judicial and extra-judicial proceedings) and loan recovery or asset disposal procedures are not burdensome or obstructive. During systemic shocks when markets experience serious signs of stress, these calculations will be significantly altered.

If a bank adopts prudent loss-provisioning policies prior to an NPL disposal or writing off an exposure, expected losses will be absorbed by loan-loss provisions and unexpected losses by the bank’s capital base. Regulations thus focus on loss provisioning against expected losses and capital for unexpected losses and expected losses that cannot be absorbed by loan-loss provisioning. Inadequate loan-loss provisioning will adversely affect bank profitability because a portion of the bank’s assets will become contra assets or an expense, eroding its capital reserves. High NPL levels weigh on bank liquidity and, in the extreme, solvency, which can disrupt financial stability and sustainable development.^9^ Systemic events can severely alter loan-loss forecasts based on the recovery expectations associated with stable markets under internal risk management, credit ratings agencies and other regulatory capital and accounting measures.

Regulators need the tools and expertise to identify rising NPL levels in order to understand bank solvency risks and to stabilise bank balance sheets. In the context of COVID-19’s dual supply and demand shocks and global economic contractions,^10^ combined and reinforced by the impact of the invasion of Ukraine, it is questionable whether bank balance sheets in a range of economies will be able to withstand the stress as the crisis evolves from a liquidity shock to a solvency risk, with high unexpected loan losses potentially leading to bank insolvencies. In the present circumstances, a financial sector crisis could easily spill over into the already severely stressed real economy. The longer the combination of COVID-19 and Ukraine economic crises lasts and while NPL levels keep rising, the risk of systemic banking crises will continue to increase particularly in commodity-importing developing countries. It follows that crisis resolution action which focuses on bank balance sheets and reduces systemic risk will greatly support both management of the crisis as well as the economic recovery. The proper management of NPLs enables banks to extend new credit, which is crucial for boosting economic activity, restoring profitability and the expeditious redemption of any bank recapitalisations.

Contemporary bank regulations and resolution regimes in the European Union (EU) and the United States (US) focus on the liability side of balance sheets. Financial Stability Board (FSB) resolution measures such as statutory and contractual bail-in mechanisms, and the building up of bail-inable debt, may prove problematic in the present economic environment. Our research suggests that the most effective approach to stabilise a banking system facing a systemic solvency crisis as a result of high NPLs, particularly caused by a systemic shock, is to transfer NPLs to an asset management company (AMC). This approach can be combined with recapitalisation mechanisms to prevent a systemic collapse and to support an economic recovery—a global objective in the wake of COVID-19 and the invasion of Ukraine.

This article analyses evidence from the three major international banking crises of the past 25 years, with examples being drawn from Asia, the US and the EU. Historical evidence supports the critical role of bank asset restructuring involving the use of AMCs, asset protection schemes and—in some cases—capital injections in achieving an expeditious restoration of banking sector health following a major macroeconomic and financial crisis. This historical analysis is supported by empirical analysis from the Bank for International Settlements.^11^

These findings indicate why the structured use of AMCs and recapitalisations—despite fears of moral hazard risk—is an effective way to manage a systemic banking crisis. The longer the COVID-19 and Ukrainian economic crises extend, the more banks will face increasing NPLs and, in some cases, will already be under stress from legacy NPLs. Pandemic and conflict-driven economic damage coupled with unprecedented supply and demand shocks, increasing levels of NPLs impairing lending support in economies worst affected by COVID-19 and Ukraine-related shocks, is threatening a systemic banking crisis across developing economies and potentially developed economies.^12^ Identifying effective bank stabilisation remedies which address the proliferation of NPLs on the asset side of bank balance sheets is of cardinal importance for avoiding a systemic banking crisis while ensuring a robust economic recovery and global financial stability.

The article is divided in eight sections. Following this introduction, Section 2 discusses internationally endorsed NPL regulatory approaches, causes of NPLs, and the economic consequences of high levels of NPLs. Section 3 examines systemic bank resolution standards which mostly focus on the liability side of bank balance sheets. Section 4 analyses the 1997 Asian Financial Crisis, focusing on the resolution approaches used in Thailand, Indonesia, South Korea and Malaysia. This includes a review of AMCs and resolution measures in China. Section 5 examines the bailouts of UBS, RBS and Citigroup during the GFC. Section 6 analyses measures adopted to stabilise the banking sector during the 2010 Eurozone Debt Crisis in Spain, Ireland, Italy and Greece. Based on our findings, Section 7 discusses recommendations and the implications for the response to COVID-19 and Ukraine. Section 8 concludes.

## Regulating Non-performing Loans: Approaches, Causes and Consequences

The first step in addressing a banking crisis is prevention, although historically prevention alone has proven insufficient. Significant work over the past 40 years has led to the development of best practices for regulation and supervision, expressed via a range of international standards formulated and implemented through the G20 and FSB.^13^ Prevention requires a range of crisis management tools including liquidity provisions and contingency planning as well as systems for the resolution of failing or failed financial institutions.^14^

Given the nexus between high NPL levels and solvency, identifying NPLs to monitor and address banking system stability is an obvious starting point, from the standpoint of prevention, yet one where there is often a surprising lack of clarity.

### Definition, Accounting Treatment and Regulatory Issues

Systemising an NPL/NPE definition is problematic because the extent of non-performance varies, resulting in different types of delinquent loans. Jurisdictions rarely share the same definition.^15^ This is explained by each jurisdiction’s banking system being unique, resulting in stylised quantitative and qualitative factors to measure NPLs/NPEs. To harmonise quantitative and qualitative criteria used for credit categorisation and for countries with no NPE definition, the Basel Committee on Banking Supervision (BCBS) released ‘Guidelines: Prudential treatment of problem assets—definitions of non-performing exposures and forbearance’. The Guidelines identify criteria to upgrade an exposure from non-performing to performing and the interaction between non-performing and forbearance.^16^

The most universally accepted method to identify credit exposures is the NPL/NPE classification. Adopting internationally endorsed NPL/NPE classifications promotes confidence in banks’ financial position, credit risk and solvency.^17^ The BCBS defines an NPE as loans and debt securities (1) defaulted under the Basel II framework, (2) that are credit-impaired according to the applicable accounting framework, and (3) all others more than 90 days due.^18^ Basel II uses a similar definition to the International Monetary Fund (IMF)—a default on principal and interest that lasts more than 90 days.^19^ Flaws in the methodology have been identified by the BCBS, notably when definitions are determined only by ex post collectability—i.e., 90 days past due.

International Financial Reporting Standard 9 (IFRS 9) provides an internationally endorsed accounting treatment for impaired assets, based on forward-looking or expected credit losses (ECLs). For developed markets, IFRS 9 officially commenced in 2018. IFRS 9 forecasts loan default losses when credit risk expectations increase. The ECL approach comprises quantitative and qualitative measures which determines the timing when a loan-loss provision is recorded and when to move an impaired asset off-balance sheet.^20^

Banks are incentivised to procrastinate over IFRS 9 impaired asset recognition that will erode capital buffers, such as bail-in triggering events. The IMF addresses the problem of impaired asset recognition being subject to banks’ discretion by recommending incentives to accelerate the transfer of NPLs/NPEs off-balance sheet.^21^ Furthermore, the BCBS supports the early recognition of credit losses. These approaches harmonise the IFRS accounting provisions with the Basel III capital requirements because any shortfalls are deducted from Common Equity Tier 1 (CET1).^22^

Accounting classifications are important because NPLs/NPEs recorded at fair value affect the level of loan-loss provisions, capital and when NPLs/NPEs are written off. Valuations are procyclical because they are overstated during periods of rapid economic expansion and understated in downturns.^23^ ECL seeks to smooth valuation volatility and strengthen banks’ capital position. The problem for ECL is that a sudden and sharp increase in NPLs/NPEs will heighten procyclicality and weaken a bank’s capital position. Thus, governments must decide whether to suspend or retain ECL accounting. If the latter is chosen, drastic measures are needed to alleviate the asset side of a balance sheet becoming inundated with NPLs/NPEs.

As a result, since March 2020, some governments have suspended IFRS 9 to delay loan-loss recognition, in conjunction with allowing banks to grant payment moratoriums to borrowers affected by the COVID-19 shutdown.^24^ The fear was that ECL accounting in the COVID-19 environment would increase procyclicality by concentrating, rather than spreading out, the reporting of NPLs/NPEs. Many individuals and businesses faced severe liquidity constraints and thus have been given additional time to replenish liquidity and remain solvent. If IFRS 9 had been retained, loan defaults and insolvencies would have increased markedly, compelling banks to use and most likely exhaust loan-loss provisions to absorb NPLs/NPEs and consequently draw upon capital buffers, possibly impacting on solvency.

### Causes of Non-performing Loans

History has shown that high levels of NPLs often arise from connected banking transactions (sometimes called *crony banking*), fraud or uncommercial underwriting standards, and contracting macroeconomic cycles that impair economic performance and devalue collateral. Contracting macroeconomic cycles pose the greatest challenge for measuring credit exposures. For example, Spain was one of the worst affected countries during the Eurozone debt crisis despite banks having sound pre-provisioning lending.^25^ Spanish real estate and the economy were disproportionately inflated by the low-interest rate policy of the European Central Bank (ECB), rendering prudential measures ineffective.^26^ This provides an important moral hazard lesson for two reasons. Spain highlights the limitations of the moral hazard argument and legislation where the macroeconomic cycle and monetary policy have caused an NPL crisis rather than bank management and shareholders (or creditors).

An insightful econometric methodology pioneered by Klein^27^ differentiates between bank-specific and macroeconomic factors using *dynamic panel* regressions. This method was adopted by the IMF to study Italian NPLs.^28^ The authors ran fixed effects and *generalised method of moments* regressions of NPLs on common macroeconomic bank variables and bank-specific variables, to determine the role each played in the build-up of NPLs. The authors found that macroeconomic variables play a significant role in the accumulation of NPLs, concluding that both bank-level and macroeconomic factors have affected Italian banks’ asset quality. Lower bank profitability is associated with higher NPL levels and a rapid loan book expansion due to high growth rates or low interest rates which, on average, results in lower asset quality:

Overall, the results show that the recession, which was of exceptional duration and intensity, had a profound impact on banks’ asset quality, which was exacerbated by bank-specific factors.^29^

### Economic Consequences of Non-performing Loans

A significant body of research suggests that banking sector NPL levels have an important influence on credit extension and growth.^30^ Weak bank balance sheets can dampen economic activity, especially in economies such as the EU which rely on bank financing. Studies have also found that banking systems with high NPLs tend to reduce credit-to-GDP ratios and GDP growth, while increasing unemployment.^31^

Aiyar et al*.* found that high NPL ratios constrain bank capital that could otherwise be used to increase lending, reduce bank profitability and raise funding costs—thereby stifling the supply of credit.^32^ Reducing NPLs is crucial to support credit growth. For this reason, the view of the European Stability Mechanism (ESM)—sole reliance on GDP growth will not lead to a substantial decline in NPL levels—is justifiable.^33^ An IMF report notes that reducing NPL levels is required for a long-term recovery following a financial crisis.^34^ While the IMF has made the NPL ratio a key measurement of financial strength,^35^ there is no explanation or definition of an acceptable NPL ratio, implying that the optimal ratio is the lowest possible. The rationale suggests NPLs on banks’ balance sheets create uncertainty and weigh on the ability to resume lending, and therefore constrain aggregate demand and investment.^36^ This uncertainty relates to a bank’s solvency^37^—not writing down the true value of NPLs—because the market presumes that the accounting value of capital is overstated. Regardless of how well a bank appears to be capitalised, NPLs reduce bank profitability, which is associated with illiquidity and insolvency.^38^

The abundance of NPLs in the EU following the Eurozone debt crisis was a significant cause of anaemic economic activity due to reduced lending and the persistent impression of bank fragility. Another unresolved issue is NPLs suppressing the economic activity of overextended borrowers,^39^ which can trap resources in unproductive activities. Resolving impaired loans is tantamount to tackling debt overhang, stimulating viable firms’ demand for new loans while encouraging non-viable firms to wind down.^40^ Unclogging bank lending channels augments the transmission of monetary policy to the real economy.

A concentration of unresolved NPLs and restricted credit supply impacts on economic growth, innovation and the Schumpeterian cycle. In the longer term, this induces unregulated or under-regulated parallel financing that can increase overall lending rather than decrease the supply of credit. A good example is China, where most legacy loans are held by state-owned enterprises operating in the manufacturing sector, in contrast to technology companies that access ingenuous and riskier (from a financial stability perspective) forms of finance. This is especially valid for NPLs generated from gyrations in the macroeconomic cycle rather than loose underwriting standards, crony banking or fraud. Thus, taking an overly principled stance vis-à-vis moral hazard in relation to NPL resolution is overwhelmingly counterproductive.

Loss recognition pursuant to IFRS 9 can influence capital buffers and trigger bail-in events. Thus, absent strong external supervision, bank management is incentivised to avoid loss recognition triggering bail-in events.^41^ For example, the IMF suggests that Italian bank managers faced a number of obstacles which disincentivised the timely resolution of NPLs.^42^ Regulatory responses in such circumstances are uncertain, in contrast to idiosyncratically resolving a single bank.^43^ This is because triggering bail-in instruments en masse could prove disruptive in a systemic crisis or a banking system excessively burdened with NPLs.^44^ In contrast, bank management intent on addressing NPLs in a timely and effective manner is key to the resumption of bank lending, tackling debt overhang, the duration and rate of NPL recovery, and mitigating bank losses. According to the IMF:

The delays depreciate the value of the NPLs, and the prices buyers are ready to pay, after discounting the delays, are not attractive for the banks. A reduction in the time to recover loans would have a positive impact in the price of NPLs.^45^

## Systemic Bank Resolutions, Moral Hazard and Liability-side Approach

Post-GFC resolution regimes, including the US Orderly Liquidation Authority^46^ and the EU Bank Recovery and Resolution Directive^47^ (BRRD), are designed to support orderly bank failures and preserve systemic stability. These regimes aim to eliminate the too-big-to-fail subsidy^48^ by curbing shareholders’ and managers’ propensity to select riskier assets,^49^ as well as include ex post mechanisms to secure adequate resources to cover bank losses.^50^

Publicly funded bank rescues are historically associated with moral hazard because senior unsecured creditors (e.g., bondholders) are typically unaffected at the expense of the taxpayer who ultimately funds public bailouts.^51^ For this reason, expectations of public bailouts are regarded as a major incentive for excessive risk taking (moral hazard) and weak monitoring by creditors. There is a widely held belief that contemporary resolution regimes can overcome this problem by eliminating public assistance or by severely curtailing access to public funds.^52^

This article argues that unlike the US and, to a large extent, the EU BRRD, bank resolution should take a more pragmatic view of this problem in finding that temporary public funding can provide the optimal approach in some circumstances, particularly when high levels of NPLs across the banking sector threaten a systemic crisis as a result of exogenous shocks such as COVID-19 or the invasion of Ukraine.

### Systemic Bank Resolutions

Effective bank resolution regimes require legal and regulatory frameworks, as well as supervision to address: (1) risk management; (2) capital and liquidity buffers; (3) large exposure restrictions; (4) transparent credit standards; (5) bank restructuring frameworks; and (6) distressed debt transfer mechanisms. From the standpoint of potential funding sources, there are numerous tools available to reduce systemic risk. For instance, globally systemically important banks (G-SIBs) which have been compared to super-polluters^53^ that spread risk due to implicit government guarantees are subject to higher loss absorbency requirements, increased going-concern loss absorbency, enhanced supervision and strong contingency planning requirements.^54^

In addition to higher capital requirements (e.g., going-concern loss absorbency), G-SIBs are required to hold total loss-absorbing capital (TLAC) as gone-concern loss absorbency. TLAC is designed to ensure funds that are available only for loss absorbency and recapitalisation for an orderly resolution to minimise financial instability, ensure the continuity of critical functions and avoid exposing taxpayers to losses.^55^ Firstly, TLAC is a precautionary measure which supports market confidence that a G-SIB has adequate resources to readily absorb losses. Secondly, TLAC can stabilise the banking system ex post, since designated liabilities can be bailed in to absorb bank losses while minimising the risk of secured creditor flight, which could certainly trigger, rather than contain, a systemic banking crisis.^56^

### Moral Hazard

Minimum TLAC must be at least 16% of the resolution group’s risk-weighted assets, scheduled to increase to a minimum of 18% in the future.^57^ These requirements are in addition to the Basel III capital requirements.^58^ Presuming that regulatory capital reflects a bank’s approach to offsetting lending and structural reforms, such as ring-fencing adopted by the United Kingdom (UK), this will render difficulties in containing moral hazard with a bail-in resolution and no public funding.

The FSB mandates that the private sector is the first funding choice for bank resolutions. Government funding conditions are designed to mitigate moral hazard.^59^ While this is a sound and principled approach, it may not be feasible in all circumstances. For example, bank rescues based on the liability/equity side may intensify market panic when a crisis is systemic and triggered from macroeconomic developments^60^ or as a consequence of exogenous factors such as the increase of NPLs from COVID-19.

Conversely, bank failures can be caused by idiosyncratic factors such as management’s focus on return-on-equity and bonuses, which can induce relaxed lending standards. In these circumstances, bailouts should be precluded because of moral hazard concerns as well as an absence of systemic risk. Creditors should also bear the full cost of bank losses once shareholder funds have been exhausted.^61^

### Liability-side Approach

The FSB Key Attributes Assessment Methodology for the Banking Sector assesses a jurisdiction’s G-SIB resolution framework compliance with the FSB Key Attributes of Effective Resolution Regimes for Financial Institutions (Key Attributes).^62^ Having bank operations in multiple jurisdictions renders cross-border cooperation, in accordance with the FSB Principles for Cross-border Effectiveness of Resolution Actions, essential to the effectiveness of a G-SIB resolution.^63^ In each jurisdiction where a G-SIB operates, the Key Attributes state that for the resolution regime to be effective

is to make feasible the resolution of financial institutions without severe systemic disruption and without exposing taxpayers to loss, while protecting vital economic functions through mechanisms which make it possible for shareholders and unsecured and uninsured creditors to absorb losses in a manner that respects the hierarchy of claims in liquidation.^64^

The options to resolve a non-viable bank are stabilisation and liquidation, which are underpinned by resolution powers.^65^ When bail-in tools are used to absorb losses by cancelling debt and strengthening equity, and therefore the liability/equity side of the balance sheet, the resolution authority’s powers encompass: (1) a write-down that respects the hierarchy of claims in liquidation, equity, or other instruments to absorb losses; (2) converting into equity or bank-under-resolution ownership instruments that respect the hierarchy of claims in liquidation; and (3) upon entry into resolution, convert or write down any bail-in instruments where terms have not been triggered.^66^

This approach explicitly provides the resolution authority with the power to sell or transfer bank liabilities and equity. In terms of the asset side of the balance sheet, these powers extend to a transfer to a bridge bank or a third-party private-sector buyer without requiring the consent of interested parties or creditors, nor constituting a contractual default or termination event.^67^ The AMC approach of selling or transferring NPLs (i.e., assets) can be an effective resolution option but it requires strengthening the regulatory powers to overcome resistance from shareholders and especially creditors (i.e., the liability/equity side of the balance sheet), given that this can inevitably crystallise bank losses and decrease investment value.

During banking crises, bank balance sheets are placed under extreme stress requiring capital injections on the liability/equity side of the balance sheet and restructuring distressed debt on the asset side of the balance sheet, including the possible sale or transfer off-balance sheet. While bank recovery and resolution measures focus on the liability/equity side of the balance sheet, the use of AMCs to restructure the asset side can also be a powerful tool in reducing costs in the long run. From this vantage, we consider the three major international banking crises of the past 25 years.

## The Asian Financial Crisis, China and the Role of Asset Management Companies

Asia experienced its most significant modern financial crisis in 1997–8. Severe economic and structural imbalances destabilised banking systems, resulting in systemic banking crises resolved through a range of regulatory approaches in Thailand, Indonesia, Malaysia and South Korea. Capital adequacy ratios of up to 10% that satisfied the requirements of the contemporary Basel I Accord (i.e., equity side of the balance sheet) proved insufficient to absorb high levels of NPLs during the Asian Financial Crisis. When banks required balance sheet and business model restructuring to remain solvent, NPL and resolution regimes were either underdeveloped or non-existent.

### Indonesia, Thailand, Malaysia and South Korea

Indonesia epitomised the policy of closing rather than restructuring banks, with bank numbers halving following state closures and takeovers.^68^ Bank closures did in fact reduce Indonesia’s NPL ratio; however, this is attributable to closing a few large banks with particularly high NPL ratios. Indonesia’s reluctance to implement reforms and promulgate legislation intensified its banking crisis and hindered NPL resolution efforts.

Eventually, Indonesia introduced asset-side measures to legally sell insolvent banks’ NPLs without needing approval from borrowers or bank owners.^69^ The remaining solvent banks were restructured by encouraging an injection of capital from new investors to partially absorb bank losses, NPLs were reorganised over 20 years, new investors pledged collateral for reorganised NPLs and remaining NPL losses were covered by a central bank loan.^70^ Over Rp400 trillion of government-issued bonds, or 35% of GDP, were issued to fund Indonesia’s bank recapitalisation programme.^71^ The government-backed Indonesia Bank Restructuring Agency was responsible for resolving Rp234 trillion of NPLs, representing 19% of GDP.^72^

Thailand’s IMF programme was designed to restructure its financial sector by identifying and closing insolvent institutions, applying blanket government depositor and creditor guarantees, and implementing structural and regulatory reforms.^73^ Nevertheless, the concentration of bank closures in Thailand did not correlate with a drop in NPL ratios over the short term. To address stubbornly high levels of NPLs, the Emergency Decree on Asset Management Company (1998) was promulgated to enable AMCs to manage distressed assets and resolve bad debts through asset restructurings, asset sales, foreclosures or litigation. Distressed debt resolution was facilitated by revised rules to allow for NPL transfers during bank restructurings.^74^ To accelerate debt restructuring, a dispute resolution mechanism was established to assist with voluntary out-of-court restructurings and to spread the debt burden between debtors and creditors. Borrowings to bail out financial institutions amounted to 1.4 trillion. Emergency legislation enabled the Thai Government to issue bonds to fund the bailouts.^75^

Resolving systemic banking crises by focusing on closures weakens confidence. Paradoxically, this was a condition of the IMF support programmes in Thailand and Indonesia. In contrast, Malaysia relied on an NPL transfer mechanism rather than bank closures. A pre-emptive crisis programme was introduced to address structural weaknesses. Malaysia’s restructuring plan fundamentally involved a merger plan, an AMC—Danaharta—to manage NPLs and recapitalisations.^76^

Danaharta was funded by the sale of government bonds (RM11.1 billion) and ceased purchasing NPLs (RM52.4 billion) in 2001 with an average recovery rate of 57% by 2002.^77^ Bank recapitalisations were managed by a government-backed special purchase vehicle—Danamodal. Malaysia’s central bank enforced Danamodal’s powers whereby capital was only injected into viable banks on commercial terms.^78^ Existing bank shareholders were decimated because all losses were absorbed by equity prior to recapitalisation. Malaysia’s fiscal backstop and NPL portfolio transfers proved successful, resulting in a more effective banking sector restructuring programme than in Indonesia and Thailand and, importantly, one that maintained confidence throughout the crisis.

An alternative approach was used in South Korea where the existing resolution framework was modified, and which proved effective in addressing NPLs. To absorb rapidly increasing NPLs, a fund was established with ₩3.5 trillion under the supervision of the Korean Asset Management Corporation (KAMCO).^79^ Viable or solvent banks’ NPLs were purchased by KAMCO on the condition of merger, management replacement and downsizing.^80^ This was supported by government capital injections and financed with bond issues.^81^ Banks with high NPL ratios were closed and weak banks had to submit rehabilitation plans.^82^ Between 1998 and 2002, nine banks merged and bank numbers fell from 33 to 19.^83^ Recapitalisations amounted to over ₩128 trillion.^84^ South Korea’s NPL ratio peaked at 8.9% in 2000 before falling to 3.4% by 2001.^85^

### Analysis and Evaluation

Weak credit and bank governance regimes, endemically lax supervision coupled with over-lending by foreign bankers placed capital markets at the heart of the domestic systemic banking crises of 1997–98. Economic collapses were reinforced by inappropriate diagnosis and conditionality from the IMF. IMF crisis management policies—reflecting the dominant consensus of the time—in Thailand, Indonesia and South Korea focused on closing and liquidating insolvent institutions, and government guarantees (i.e., asset side of the balance sheet). Capital (i.e., equity side) restructuring was viewed as the last resort. Nonetheless, Indonesia and Thailand had the highest level of closures and experienced the deepest and longest disruptions to their banking systems and the most extensive use of public funds. Despite this starting point, radical asset-side balance sheet restructuring undertaken and supported by public funds reduced taxpayer exposure and ex-post bank losses. Asset-side restructurings enabled a resumption of lending and restoration of financial stability despite initial errors by domestic and international authorities in the context of blanket guarantees, nationalisations, sovereign solvency and currency crises.

The East Asian experience shows that asset-side debt restructuring and legal frameworks, rather than bank closures, proved to be the most effective approach to revive banking system health. All resolution programmes involved public funding, although there were variations in the approach to restructuring. Government guarantees were critical for stabilising banking systems and a condition of IMF bailouts. The use of AMCs was instrumental in cleansing balance sheets of NPLs, strengthening capital ratios and re-starting lending to aid the economic recovery. AMCs were funded either by government capital injections or by the sale of bonds. Legal and regulatory infrastructure was a prerequisite for the expeditious transfer and sale of NPLs. In our view, these are important findings that support a strong asset-side approach to balance sheet restructuring to supplement existing bank crisis management and resolution regimes.

### China

Despite strong GDP growth, China’s banking system in the late 1990s was characterised by structural weaknesses, nascent prudential supervision, and lax underwriting standards. In 1997, China’s NPL ratio was 20%.^86^ Reforms to address NPLs involved the recapitalisation of state-owned banks, adopting international NPL classification standards, enforcing commercially viable loans, and banning local governments from influencing lending decisions.^87^ The last two reforms centred on strengthening credit standards and quashing connected lending. Similar to the banking systems most impacted by the Asian Financial Crisis, China’s bank recapitalisations were funded extensively by government bonds, approximately RMB270 billion.^88^ This liability-side approach was not effective in addressing what in reality was the creation of a commercial banking sector from the previous mono-bank system, essentially one in which budget transfers were initially labelled as *loans*, with no expectation of repayment. It was thus a fundamentally different problem than that faced by countries at the heart of the Asian Financial Crisis, of which China was not one due to its strict controls on both capital and the financial sector.

In 1999, China embraced an asset-side approach by establishing four state-owned AMCs to transfer NPLs from corresponding state-owned banks.^89^ NPLs were purchased by state-owned AMCs issuing bonds, with credit supplied by the central bank. Disposals were slow and the recovery rate was 21%, reflecting the underlying reality that many of the NPLs had been in fact budgetary transfers to state-owned enterprises.^90^ Although NPL ratios eventually fell to 2.4% by 2008, the reduction was attributed to extremely strong GDP growth, rather than NPL transfers to the AMCs.^91^ China thus eliminated the legacy of its earlier financial system through a combination of time, growth and restructuring.

As China’s growth rates decelerated over the past decade in the wake of the 2008–9 GFC and levels of indebtedness rose, NPLs have substantially increased, reaching $1.5 trillion by June 2019.^92^ Prior to May 2019 bank bailouts were rare in China. This changed when the People’s Bank of China (PBOC) and the China Banking and Insurance Regulatory Commission (CBIRC) decided to nationalise the Bank of Baoshang. Since then and with the onset of COVID-19, several local and provincial banks have been either bailed out or closed.^93^ To curb this increasing fiscal burden on the national government, the PBOC stated in September 2019 that shareholders would be primarily responsible for future bank failures.^94^ However, this does not necessarily equate to the use of private sector funding. For example, in April 2020 the Bank of Gansu underwent a restructuring whereby equity holders would raise their stake from 28% to almost 50%. Equity holders in this instance were state-backed.^95^

The CBIRC relaxed NPL recognition rules in February 2020 when the economic ramifications from the COVID-19 pandemic became apparent.^96^ This is contrary to IMF guidance on preserving financial stability, maintaining banking system soundness and sustaining economic activity during the COVID-19 pandemic: ‘Loan classification and provisioning rules should not be eased, and it is critical to measure NPLs and potential losses as accurately as possible.’^97^ Nonetheless, China’s economy, financial system and NPL ecosystem are quite different to 20 years ago and are more capable of handling large volumes of NPLs. The big four banks are not the primary source of NPLs and systemic risk, as was the case 20 years ago. Small- and medium-sized banks (i.e., local and rural) are the biggest source of systemic risk with high levels of poor-quality NPLs that collectively form a large segment of the banking system.^98^ COVID-19 is proving to be a serious test to China’s NPL ecosystem and its bank restructuring policies, in particular its lack of a formal bank resolution framework.

## The Global Financial Crisis and Bank Rescues: Pragmatism over Ideology

During the GFC, major economies adopted a range of approaches to address systemic banking solvency crises which contrasted strongly with initial IMF-mandated responses in Asia in 1997, with leading examples in Switzerland, the UK and US to restructure UBS, the Royal Bank of Scotland (RBS) and Citigroup in 2008. Switzerland and the UK employed Asset Protection Schemes (APSs) that utilised state guarantees and capital support rather than distressed asset sales. The US opted for a guarantee and the Troubled Asset Relief Program (TARP).^99^

In the early stages of the GFC, bailouts of G-SIBs were preferred to closure and liquidation because of the lack of legally viable resolution tools, appropriate contingency planning, and out of caution to avoid a systemic crisis, as eventually occurred with the failure of Lehman Brothers. In contrast to 1997, in the GFC, major developed country governments provided massive capital injections to prevent bank closures and liquidations. The introduction of APSs generally avoided the need for bank nationalisations and thus banks or distressed assets being placed on government balance sheets.

For example, UBS received a government capital injection of CHF6 billion from the central bank, the Swiss National Bank, consisting of mandatory convertible notes (i.e., converting into equity), to enable the sale of NPLs and NPL-linked instruments.^100^ These distressed assets were then transferred to a special purpose vehicle (SPV) analogous to an AMC: the StabFund.^101^ In the UK, the government was forced to purchase most of RBS’ shares due to a dearth of private sector buyers, effectively nationalising RBS but not sufficiently so to achieve consolidation. Bank of England (BoE) emergency loans provided an additional £20 billion recapitalisation.^102^ The government held 90.6 billion shares or 84% of RBS’ capital.^103^ A condition of the recapitalisation was participation in the Asset Protection Scheme, which was established to protect banks against losses on distressed assets.^104^ RBS also sought protection for £282 billion in distressed assets including NPLs.

In the US, the $700 billion TARP was introduced to purchase distressed assets but was eventually used for mandatory government recapitalisations.^105^ Citigroup alone received $25 billion as part of TARP and, on 23 November 2008, agreed to a government bailout which included a $301 billion government guarantee on a pool of distressed assets under the Asset Guarantee Program. Distressed assets were retained on Citigroup’s balance sheet.

Each G-SIB was facing insolvency from excessive leverage and insufficient capital levels. Losses mounted and capital was exhausted through losses from exposures to complex products, as a result of changes in both the property and economic cycles, capital market closures for non-government debt, rating downgrades from Basel II requirements and fire sales to meet capital shortfalls. Distressed assets complicated and inhibited the use of AMCs for sequestration purposes. RBS and Citigroup were subject to government guarantees and retained distressed assets on-balance sheet. In contrast, UBS transferred distressed assets to an AMC—a similar process to that adopted in the Asian Financial Crisis. Switzerland injected capital and took an ownership position in UBS at the beginning of its programme. Both approaches strengthened bank balance sheets and stabilised banking systems, eventually enabling the financial system to return to stability, even though governments were exposed to extensive bailout funding risk.

The Swiss and UK rescue frameworks were premised on existing legislation to facilitate prompt implementation, in contrast to the US TARP (new legislation, initially rejected) used in tandem with the existing Emergency Economic Stabilization Act of 2008. Participating banks signed contractual agreements with regulators to facilitate restructuring and to be held accountable. An initial hesitation by RBS subsequently forced the UK government to purchase equity after a share issue failed.

These two restructuring approaches highlight the advantage of controlling the realisation of asset values when using AMCs. The guarantee schemes were nonetheless profitable in most cases, relatively short lived despite substantial taxpayer funding risk and particularly effective in stabilising G-SIBs, stemming creditor runs and maintaining banking system stability. The key difference between these approaches is that under a guarantee scheme distressed asset sales are usually realised when market conditions are depressed, and losses are greatest. AMCs can control and prolong the timing of asset value realisation by selling NPLs in favourable market conditions, thereby mitigating losses and taxpayer funding risk.

## The Eurozone Debt Crisis and Banking System Restructuring

Before analysing the impact of the Eurozone debt crisis on the banking systems of Spain, Ireland, Italy and Greece, we examine the post-2014 Single Resolution Mechanism (SRM), the BRRD and the post-2018 bank debt restructuring regime. From our analysis, one point stands out: Eurozone countries were more proactive in addressing banks’ distressed debt before the implementation of the BRRD, even though the EU state aid regime has remained largely intact.

### European Union Resolution Regime

The Single Supervisory Mechanism (SSM) was the first step towards an EU banking union applicable to member states’ banks.^106^ Its main aims are to ensure safety and soundness of the EU banking system, increase financial integration and stability, and ensure consistent supervision.^107^ Another pillar of the EU banking union is the SRM. The Single Resolution Board (SRB) in conjunction with the national resolution authorities form the SRM, which is designed to ensure an orderly resolution of banks while mitigating taxpayer expenditure.

In 2014, the EU enacted the BRRD, which largely conforms with the Key Attributes, to deal with failing banks as opposed to the use of national regimes.^108^ The paramount purpose of the BRRD is to eliminate public bailouts and thus contain the *doom loop* which can bind together sovereign and banking sector solvency—so clearly seen in Asia in 1997 and in Europe in 2008 and 2010. This avoids the mutualisation of bank risk in the Eurozone by supressing the fiscal burden sharing of bank losses among EU members.^109^ A BRRD resolution must satisfy a number of objectives: (1) safeguarding the continuity of essential banking operations; (2) protecting deposits, client assets and public funds; (3) minimising risks to financial stability; and (4) avoiding unnecessary destruction of value.^110^

Part IV of the BRRD specifies four resolution tools: (1) sale of business; (2) bridge institution; (3) asset separation (i.e., AMCs); and (4) bail-in.^111^ Bail-in tools are viewed as important to mitigate moral hazard when there is a strong reliance on bailouts. The BRRD bail-in tool focuses on the liability/equity side of the balance sheet by allowing the resolution authority to write down or convert the claims of creditors to equity, in accordance with a predetermined hierarchy. This reduces the extent of a capital injection (and the taxpayer burden) which in principle acts as an additional capital buffer.^112^ The BRRD requirement for a minimum bail-in of 8% of liabilities before any contribution of public funds, or from the resolution fund, is viewed as a big hurdle for a bank in resolution.^113^

In July 2017, the EU Economic and Financial Affairs Council issued an action plan to move NPLs off-balance sheet and establish member state AMCs.^114^ At the time there were almost €1 trillion NPLs and of that, small and medium enterprises constituted the largest proportion.^115^ The 2017 NPL action plan outlines: 116

(i) more intensive supervision for banks with high levels of NPLs;
(ii) reform of domestic insolvency and debt recovery frameworks;
(iii) development of secondary markets for distressed debt (i.e., NPLs); and
(iv) the use of private-sector AMCs to provide a structural solution for distressed debt markets.

By March 2018 the European Commission had submitted a package of measures together with the Second Progress Report on the Reduction of NPLs in Europe.^117^ The European Parliament and Council endorsed the 2018 NPL proposals by agreeing, in June 2019, to pass the *banking package* into EU law with the promulgation of the Capital Requirements Regulation (CRR II)^118^ and the Capital Requirements Directive. In April 2019, amendments to the CRR II created a statutory prudential *backstop* which is designed to prevent under-provisioning for expected loss NPLs.^119^

The objective of these measures is to reduce NPL ratios and future excessive NPL accumulations. These measures are taxonomised as follows:

(i) augmenting market-based solutions for the massive disposal of NPLs through legal and regulatory reforms and EU-wide infrastructure; 120
(ii) building a liquid market for distressed debt, at the domestic and EU level; 121
(iii) expanding the micro-prudential framework through supervisory requirements imposed by the SSM. Firstly, the timely detection and effective management of NPLs. Secondly, establishing quantitative NPL reduction targets.^122^

To achieve these objectives, banks will improve NPL governance and use NPL reduction approaches as described in the ECB Guidance to Banks on NPLs (2017).^123^ Banks are to introduce hold/forbearance strategies that, depending on borrower capability and expertise, can lead to workouts and active portfolio reductions through sales and writing off provisioned NPL exposures that are deemed unrecoverable, or change the type of exposure through foreclosure, debt-to-equity swaps, debt-to-asset swaps or collateral substitution. Moreover, banks are required to hold prudential backstops to compel provisions for NPLs ex ante and thus have adequate capital reserves when writing off NPLs.^124^ This is a proactive measure that targets future accumulation of NPLs by incentivising banks to take ex ante action against NPL accumulation.^125^

The European Commission has published blueprints on how to develop private-sector AMCs^126^ and on a liquid pan-European market for distressed bank debt, exclusive of state support.^127^ These market-based solutions are expected to be supported by the future introduction of legislation, in accordance with the EU 2019 *banking package* on the liquidation of collateral.

### The Eurozone Banking Crisis: Asset Management Companies and Guarantees

#### Spain

Spain experienced a property bubble prior to the Eurozone debt crisis. After the bubble burst in January 2009, Spain entered recession at which point NPLs exceeded 4%.^128^ The government established the Fund for Orderly Bank Restructuring (FROB) to restructure banks. FROB was capitalised with €9 billion to take over non-viable banks, subscribe convertible instruments to merge viable banks and subscribe ordinary shares to recapitalise viable banks.^129^ The banking system reform strategy was implemented in three phases: consolidation, solvency improvement and cleaning up balance sheets.^130^

Following a second recession in 2012, Spain sought a banking system bailout of €100 billion from the ESM. Financial assistance was implemented through FROB in accordance with EU state aid rules.^131^ The bailout programme consisted of early intervention, restructuring and resolution.

Banking system stress tests identified deficient capital levels which resulted in €38.9 billion of partial bank nationalisations and €2.5 billion to establish the Asset Management Company for Assets Arising from Bank Restructuring (Sareb).^132^ SIBs own 55% of Sareb while FROB (i.e., the government) owns 45%.^133^ FROB has the power to transfer distressed assets from banks to Sareb for independent management.^134^ Sareb’s purpose was to receive, manage and dispose of distressed assets from banks in receipt of government assistance.^135^ In exchange for distressed assets, Sareb issued government guaranteed bonds that can be used as collateral for financing (similar to the approach adopted by China in the 1990s).^136^

From January 2013, banks were required to hold a capital ratio of 9%.^137^ Banking system NPLs at the time were about €330 billion.^138^ Spain exited the EU financial assistance programme in January 2014. The NPL ratio rose to 9.4% in 2014 before dropping to 5.5% in 2016, and to 3.1% by 2019.^139^ Although Sareb has been successful in reducing banks’ NPL ratios to manageable levels, it has posted losses for every financial year since its inception. In principle, an NPL *self-cure* is critical to Spain’s recovery and Sareb’s profitability because 100% of Sareb’s assets were held in Spain and collateralised in real estate. A depressed property market and market competition have contributed to Sareb’s losses.

#### Ireland

Ireland is one of the best examples of a successful implementation of a state-backed AMC. The National Asset Management Agency (NAMA), established in December 2009, fully repaid €31.8 billion of total debt by March 2020 and was expected to post a €4 billion surplus prior to the advent of COVID-19.^140^ This was achieved despite NAMA having bought the bulk of its NPLs at a premium over market price, based on the principle of so-called Long-Term Economic Value (LTEV).

The chronicle of NAMA unfolded as follows. Ireland experienced a credit boom typified by connected lending and low credit standards that produced a highly levered banking system heavily exposed to the property market.^141^ Illiquid wholesale funding markets coincided with a downturn in the credit and property cycles, triggering a classic property-led banking solvency crisis.^142^ The purpose of NAMA was to address serious economic threats and the stability of banks and the finance sector by, *inter alia*: (1) producing an expeditious and efficient economic recovery; (2) protecting state and taxpayer interests; (3) restructuring banks; and (4) restoring banking system confidence.^143^

In December 2010, Ireland—following one of the few developed economy outright bank nationalisations of the 2008 Global Financial Crisis which transformed a private sector banking solvency crisis into a sovereign solvency crisis in much the same way as had occurred in Thailand in 1997—accepted an IMF/EU €85 billion bailout. Key objectives of the rescue programme were—very different to those of 1997 and reflecting lessons learned by the IMF as a result of those experiences—to identify viable banks and implement strengthening measures (i.e., downsizing and reorganisation), recapitalise banks, encourage bank deposit inflows and market-based funding, strengthen banking supervision and introduce a bank resolution framework.^144^

NAMA acquired bank NPLs secured on real estate amounting to €74.2 billion, involving 850 debtors and 11,000 loans collateralised on 16,000 properties.^145^ NPLs were acquired at a 57% discount over face value and below book value, yet above market value due to the LTEV premium. NAMA paid for the purchase of NPLs by issuing government guaranteed senior notes and, to a much lesser extent, subordinated debt securities.^146^

Delays in restructuring distressed debt included legal obstacles, such as a 1-year foreclosure moratorium on defaults and a High Court decision to prohibit summary proceedings for mortgages originating before 2009.^147^ In October 2017, all senior debt had been redeemed (3 years ahead of schedule) and in March 2020, all subordinated debt was redeemed.^148^

Although Ireland exited the IMF/EU bailout in December 2013, Irish banks still held a substantial volume of NPLs on-balance sheet. NPLs peaked in 2013 at 25%, more than 2 years after transfers to NAMA began.^149^ In 2014, the NPL ratios for the three largest Irish banks were 17%, 33% and 45%.^150^

From 2013 to 2017, the volume of NPLs on bank balance sheet fell from €80 to €30 billion. This reduction is not solely attributable to NAMA because two thirds of the 2017 NPLs were derived from house purchases. Banks’ mortgage books have experienced a *self-cure* because of improved economic conditions—as often happens with global banks’ credit default obligation portfolios.^151^ Ireland’s NPL ratio fell to 20% in 2014, 15% in 2016 and 5.7% by 2018.^152^

#### Italy

The Italian economy prior to 2008 experienced a prolonged period of low growth because of structural economic imbalances and an inert public sector. With the onset of the Eurozone debt crisis in early 2010, credit conditions tightened when wholesale funding markets became illiquid and credit risk intensified. By the end of 2011, the Italian banking system’s CET1 averaged 9.3% with leverage lower than comparable European banks.^153^ Italy’s NPL ratio was 11.7% with over half of gross NPLs being bad debts.^154^ This is attributed to a banking system comprising many small banks that are inexperienced in managing NPLs and a deficiency of mechanisms to efficiently dispose of NPLs.^155^ The low-growth environment and Eurozone debt crisis contributed to Italy’s very high levels of sovereign indebtedness, which hovered around 135% of GDP between 2014 and 2020 before surpassing 150%.

In November 2015, four unviable small banks were recapitalised by the central bank’s AMC, the National Resolution Fund, which in turn was financed with €3.6 billion of contributions from the Italian banking sector. Existing shareholders and subordinated debtholders absorbed losses.^156^ All four banks were restructured into bridge banks with bad debts transferred to an AMC.^157^ The European Commission approved a sale of three bridge banks to UBI Banca for the nominal consideration of €1, because each was burdened with high levels of NPLs, requiring a total of €450 million in capital.^158^ A condition of the sale obliged the National Resolution Fund to make an €810 million capital injection and grant risk guarantees.

In 2016, Italy sought to bail out Monte dei Paschi di Siena (MPS), Italy’s third largest bank, by capitalisation, after it failed the European Banking Authority stress test and was subsequently unable to raise sufficient capital. In July 2017, the European Commission approved an €8.1 billion recapitalisation on the condition of transferring NPLs off-balance sheet and capping executive pay. Concerns were raised by the ECB over MPS’ ability to maintain capital buffers. The Italian government responded by underwriting a €3.9 billion capital injection and converted €4.3 billion of subordinated junior bonds to equity, resulting in the state acquiring a 70% ownership stake.^159^ Italy designed the recapitalisation to circumvent the BRRD in order to fully compensate retail junior (bailed-in) bondholders, who had been subject to mis-selling, by exchanging €1.5 billion of their bailed-in equity with newly issued senior bonds.^160^ The capital injection and compensation to retail junior bondholders amounted to €5.4 billion in net public funding. In May 2017, two banks were liquidated as precautionary recapitalisations were not deemed viable by the ECB and the SRB. Shareholders and junior bondholders shared losses and no bail-in mechanism was used.^161^

In February 2016, the Ministry of Economics and Finance issued a securitisation guarantee (GACS) that is essentially an APS for senior notes issued by SPV purchasers of NPLs. Access to GACS is contingent on paying a fee and large banks have set up AMCs to dispose of NPLs off-balance sheet.^162^ Banks are incentivised to transfer NPLs off-balance sheet because the guarantee effects a true sale, reduces risk and uncertainty, and ameliorates price discovery. Initial NPL transfers were relatively low until 2017 when a few enormous NPL sales were finalised by Italy’s largest banks. GACS has been instrumental in transferring large volumes of NPLs off-balance sheet and has significantly reduced banks’ NPL ratios. Italy’s NPL ratio dropped sharply from 17% in 2016 to 6.7% by 2019.^163^

Large banks, hedge funds and private equity firms have formed SPV partnerships targeting corporate loans. These partnerships restructure companies with, for instance, debt-to-equity swaps and capital injections.^164^ For example, KKR Credit launched an AMC called Pillarstone Italy in October 2015. Pillarstone has two functions: NPL resolution and corporate restructuring.^165^ Companies are relaunched after Pillarstone injects capital and absorbs NPLs sourced from Italian banks.^166^

#### Greece

Doubts concerning the sustainability of Greek debt became apparent in the second half of 2009 as the economy entered recession and a sovereign debt crisis unfolded. Investors began to lose confidence in Greece’s ability to service its bonds. In April 2010, the Greek government requested an IMF/EU bailout. In contrast to Ireland, Greece was a classic example of a sovereign solvency crisis where banking sector problems resulted from its economic collapse.

Conditions of the €110 billion package included reining in fiscal spending, structural reforms to rebalance the economy and stabilising the banking system by, *inter alia*, establishing the Hellenic Financial Stability Fund (HFSF)—a private legal entity where the government is its agent under the aegis of EU member states.^167^ Banks maintained liquidity and capital via the HFSF and ECB Emergency Liquidity Assistance (ELA). These arrangements assisted in bank reconstructions, providing loans for resolutions and managing NPLs.^168^

Twelve banks were placed into liquidation or resolved during 2013.^169^ The following year, four of the largest SIBs—Eurobank Ergasias, NBG, Alpha Bank and Piraeus Bank—were recapitalised by HFSF which became a majority equity holder in each bank. NPLs were retained on-balance sheet as a distressed debt legal framework did not become operational until November 2015. By 2016, the NPL ratio reached 47% before easing to 36% in 2019, the second highest in the Eurozone.^170^

On 17 May 2016, KKR Credit reached an agreement to assign and manage the credit and equity exposures in an AMC managed by Pillarstone.^171^ In contrast to Pillarstone Italy, the European Bank for Reconstruction and Development (EBRD) has provided a capital injection of up to €50 million and Pillarstone Greece offers corporate governance advice.^172^ Pillarstone Greece was the first SPV licensed by the Bank of Greece to manage NPEs.

In late 2019, the Greek government launched an APS analogous to the Italian GACS: the Hercules Asset Protection Scheme (Hercules). Banks pay a fee for a securitisation guarantee of senior notes issued by SPVs that are recipients of their NPEs. Hercules was conceived to remove €30 billion worth of NPEs from banks’ balance sheets by 2021.^173^ These NPE reduction targets have been placed in serious doubt considering that Greece is one of the worse affected economies in the EU from the COVID-19 pandemic.^174^

### Analysis and Evaluation

Spain, Ireland and Greece merged and nationalised (i.e., recapitalised) banks prior to establishing AMCs. Closures and liquidations were viewed as the last resort. Capital injections have been critical in maintaining bank solvency and stability. When the property markets in Spain and Ireland collapsed, NPL ratios rose significantly, mirroring Thailand and Indonesia in 1997. The 2006 NPL ratios in Spain and Ireland were less than 1%^175^ because of the 2005 adoption of incurred loss accounting standards and securitisation which allowed banks to reduce loss provisioning.^176^ Italy, which used the same standard, had an NPL ratio of 6.6% in 2006, higher than South Korea and Malaysia, but significantly lower than Indonesia and Thailand.^177^ Similarly, Greece’s NPL ratio was 4.6% in 2008 under the same standard.^178^ This is alarming because NPLs were clearly understated. It follows that incurred loss accounting should be avoided during times when markets are stable. A better approach is the use of banking system stress tests, which in Spain (2012) identified the need for additional capital requirements.^179^

Spanish state aid involved diagnosing bank capital requirements based on asset quality, transferring distressed assets to an AMC, recapitalising and restructuring viable banks and an orderly resolution of non-viable banks involving burden sharing with the private sector.^180^ In contrast, Ireland took a consolidated approach with NAMA, which purchased NPLs and provided capital, credit and undertook restructurings and reorganisations.^181^ Ireland established NAMA prior to its EU/IMF bailout, similar to Malaysia in the 1990s, which assisted in stabilising its banking system.

In Italy, private-sector SPV partnerships circumvented inefficient NPL legal procedures.^182^ Following the introduction of legislative reforms, progress was initially slow because Italy’s distressed asset market was virtually non-existent prior to 2015.^183^ This has since improved markedly with the NPL sale and transfer framework working efficiently and effectively. A decree was issued by the Italian government in June 2017 that provides the legal framework for bank liquidations, including public support to guarantee an orderly exit from the banking system.

Bond issues funded the purchases of NPLs from banks in Ireland, Spain, Italy and Greece.

Lessons learned from Irish and Spanish public-private AMCs suggest that the efficient use of resources by an AMC is contingent on: (1) the development of the collateral market (e.g., real estate); (2) collateral concentration; (3) NPL quality; (4) the level of market competition; and (5) foreign investor participation.

Delays in establishing legal frameworks to facilitate efficient NPL transfers destabilised the Italian and Greek banking systems. Prior to legal reforms in Italy, one third of credit recovery procedures lasted between 3 and 5 years^184^ which kept NPLs at excessively high levels.^185^ Following successive bank recapitalisations and the promulgation of NPL laws to facilitate AMC transfers, Italy and Greece have reached agreements with private-sector AMCs to facilitate asset transfers off-balance sheet. Legislation per se is not sufficient to reduce NPL levels with the use of AMCs, as viable AMCs require well-functioning distressed asset markets.^186^ Successful distressed asset markets are, in turn, characterised by short legal processes.^187^ Evidence suggests that domestic markets for distressed assets grow in tandem with the level of NPLs, viable AMCs and expeditious transfer and sale mechanisms.^188^ For structural reasons, the EU market for distressed debt is relatively illiquid. Eliminating or diminishing the profit incentive for NPL purchases produces a disincentive for AMCs to participate in distressed asset markets, which constrains market development and liquidity.

The use of wholly private-sector AMCs in Italy is proving to be a profitable and effective asset-side approach, with strong market growth.

Italy’s GACS incentivises banks to transfer NPLs because the guarantee increases prices. AMCs are incentivised to purchase Italian NPLs because securitised notes are guaranteed at investment grade, lowering their funding costs. Greece’s Hercules differs from GACS as senior notes are not investment grade. This arrangement increases funding costs and therefore reduces profitability and the scope of NPL purchases. Government guarantees require calibration to balance the competing incentives of NPL transfers off-balance sheet and the NPL purchases by AMCs.

In the reality of the COVID-19 pandemic, the utilisation of state-backed AMCs will depend on the bargaining power of member states with the EU and the volume of new NPLs. Member states with fragile banking systems will introduce state-backed AMCs, such as Greece, to manage the COVID-19 surge of NPLs. This prediction is relevant given our survey of AMC performance in the EU during the early stages of the GFC and the Eurozone debt crisis.

## Recommendations and Responses to Macroeconomic and Sustainability Shocks

### Recommendations and Limitations

When there is the threat of a systemic banking crisis due to a surge of NPLs, asset-side restructuring has often been an effective approach for maintaining banking system viability and stability in developed and developing countries. Conditions underpinning an effective asset-side approach centre on legal and regulatory infrastructure, AMC funding and asset valuations.

Effective legal and regulatory systems are vital for the expeditious transfer and sale of NPLs to maintain banking system viability. Our research has shown that developed and developing countries which have used ex ante legal and financial infrastructure were able to efficiently transfer NPLs off-balance sheet, stabilise banking systems, mitigate economic damage and accelerate the recovery. Legal infrastructure augments the massive disposal of NPLs. If the NPL market is underdeveloped or obstructed, the government needs to introduce policies that create investment incentives or remove legal and regulatory obstacles. Without this framework, credit recovery procedures can last for years which will prolong the economic and banking system recovery.

Effective legal and regulatory systems have civil procedures that expedite court hearings; arbitration laws that facilitate negotiations between debtors, creditors and banks; bankruptcy laws that allow for foreclosure and out-of-court workout procedures; bank resolution laws to enable balance sheet restructuring, and the legal transfer, sale and assignment of NPLs; and tax laws that do not penalise NPL sales. Legal systems should enable all banks, regardless of size, to participate in the restructuring programme. The risk for many countries is that one or more of these legal prerequisites will be neglected, producing a legal obstacle for the effective disposal of NPLs.

For developing countries, viable NPL sales require a stable and growing economy, not an economy subject to capital flight and a deficit of international confidence. Unresolved NPLs and a restricted credit supply can constrain economic growth and the business cycle. Non-performing loans will begin to be generated from gyrations in the macroeconomic cycle in addition to those from the banking crisis. Developed countries are less likely to experience capital flight, and international uncertainty has historically been transitory when compared to developing countries. The primary risks for a viable NPL market in developed and developing countries are underdeveloped legal systems or unprofitable NPL sales.

Despite adverse macroeconomic conditions in developed countries, when the supply of distressed debt is limited, the sale of NPLs can become commercially viable, for example, in the US and EU during the COVID-19 shutdown. This was attributed to deferred insolvencies, unprecedented demand from special situations funds, extensive central bank market liquidity programmes, and the crisis being economic rather than emanating from the banking system.^189^

We recommend that, when possible, the majority of AMC funding be sourced from the private sector (i.e., bond issues), because this will act as a counter-cyclical relief mechanism that stabilises a banking system overly burdened with NPLs, mitigating taxpayer expenditure. However, this is rarely possible in banking crises. Thus, public funding should not be ruled out. Despite short- to medium-term credit risks, a well-designed publicly funded approach can be the most effective and efficient solution in the longer term when private sector funding is unavailable, because the government can stabilise the banking system by delaying NPL sales until economic conditions are favourable and credit growth has resumed.

However, government guarantees and publicly funded AMCs place the upfront cost and credit risk on the taxpayer, not on banks or the private sector. To incentivise private sector funding, governments should introduce guarantees enforced by contract, as a component of the NPL transfer mechanism pursuant to bank resolution laws. Publicly funded AMCs should be established by legislation that outlines the role, responsibilities, liability, business model and life cycle of the entity.

The valuation of NPLs is critical to the transfer mechanism. If assets cannot be bought at a discount to purchase and holding costs, then the asset sale mechanism and AMC will be unviable without government support. When NPLs are retained on banks’ balance sheets, there is a risk that the NPL sale price will be unprofitable because of the pressing need to relieve balance sheet stress. This can result in a loss of control over the sale which can occur at the least profitable time, for example, during a financial market collapse. Banks that have a substantial NPL exposure retained on-balance sheet will require governments to inject large amounts of capital to maintain solvency, exposing taxpayers to loss. For these reasons we recommend that NPLs be transferred off-balance sheet to an AMC.

Accounting rules determine the metrics for valuations based on either incurred or expected loss standards. Both metrics can become pro-cyclical in the right environment. Incurred loss accounting underestimates exposures leading into a banking crisis. For example, following a period of loose lending standards which has fuelled a speculative bubble where collateral markets have collapsed, incurred losses can rapidly escalate to drain a bank’s liquidity and capital reserves. In the aftermath of the GFC, governments identified incurred loss accounting as a major cause of the banking system collapse.

Expected loss accounting can overstate losses at the start of an economic crisis. This will constrain the extension of credit at a time when it is most needed, for example, during the COVID-19 economic shutdown at the beginning of 2020. Consequently, governments retained incurred loss accounting because projected short- and medium-term losses would have been amplified and bank balance sheets overly stressed under expected loss accounting (e.g., IFRS 9). COVID-19 is, however, a different situation, because the crisis did not originate in the banking system or credit markets, as is the invasion of Ukraine. Expected loss accounting is nonetheless recommended because it is designed to support balance sheet stability going into a banking crisis, through loss provisioning to absorb future NPLs. COVID-19 has shown that supervisors must exercise discretion when deciding on the appropriate accounting standard that does not induce procyclicality leading into a financial or economic crisis.

### Responding to Macroeconomic and Sustainability Shocks: COVID-19 and Ukraine

The economic fallout from COVID-19 has been contingent on countries inoculating the wider public and opening up their economies. This is particularly challenging for developing countries which are struggling to obtain sufficient quantities of vaccines. In response to the economic shutdown, many governments legislated to grant forbearance and foreclosure protection. Temporary repayment moratoria support aggregate NPL ratios remaining stable.^190^ The premise for these measures is that when an economy reopens, borrowers will be in a stronger financial position to meet their repayment obligations. For example, US unemployment fell to 3.9% and nominal GDP reached 33% in Q3 before settling to 6.9% in Q4 of 2021.^191^ Pre-COVID Ireland underlies this premise because a large part of its recovery was attributed to NPLs *self-healing* from a strong macroeconomic environment. During 2022, while the economic impact in developed and major economies has largely been addressed, inflation emerged as a major concern, with significant risks arising from rapid increases to interest rates from major central banks. The invasion of Ukraine in early 2022—as a new supply and inflationary shock—has raised risks in developed but also in particular developing country and emerging market financial systems.

Developing countries are facing a much weaker economic recovery, massive debt and equity outflows, and rapidly rising sovereign debt.^192^ In April 2021, the IMF allocated US$274 billion in Special Drawing Rights to developing countries with large sovereign debt burdens. This is a vital macroeconomic monetary stabilisation measure that boosts currency reserves by providing liquidity support. Special Drawing Rights cannot, however, be used to pay down sovereign debt.^193^ In comparison to developed countries, the macroeconomic fallout from COVID-19 in developing countries is severely hampering the recovery and banks’ ability to address debt overhang. This situation has been dramatically worsened in the context of commodity-importing developing countries as a result of the Ukraine crisis and throughout emerging economies and developing countries more generally as a result of inflation and interest rate increases. If this fragile economic and financial environment is not sufficiently stabilised, it could provide the catalyst for a major developing country banking crisis. It is therefore paramount that developing countries implement a legal framework that promotes an asset-side approach to bank balance sheet restructuring which is supported by the IMF funding government-backed bank stabilisation measures.

Another important measure to assuage stressed balance sheets is legally empowering banks and distressed borrowers to renegotiate loan terms through out-of-court workouts. For other distressed borrowers, repayment moratoria are merely delaying the recognition of NPLs which are reported on-balance sheet when the moratoria expire. In developing countries, there will be a surge of NPLs from the moratoria delaying the normal cycle of distressed debt and the increase of NPLs from the COVID-19 shutdown. A strong asset-side response will be necessary so that banks can continue to support the economic recovery when this NPL surge begins to surface.

In developed countries that are having a strong rebound in economic growth and rising market values with low debt overhang, some NPLs will *self-heal* while others will be readily absorbed into the expanding economy, expediting the off-balance sheet process to strengthen the banking system. In developing countries which cannot fund economic stimulus, the macroeconomic decline combined with the lingering pandemic will erode international investor confidence, impeding the viability of the NPL transfer process.

## Conclusion

Evidence from experience over the past 25 years supports the use of public funds where private sector funding is unavailable and the bank rescue programme involves a radical asset restructuring of balance sheets, particularly in the context of SIBs. Robust capital, leverage and liquidity buffers reduce the risk of bank failures. However, regulators can misjudge banking system strength by relying on compliance with international standards that overly focus on the equity/liability side of the balance sheet. Banks that are fully compliant (ex ante) with international standards can experience a rapid deterioration of their capital position from exogenous and endogenous shocks, namely adverse macroeconomic developments or contagion from a financial crisis. When a bank is under severe stress from systemic and macroeconomic factors, the argument against public support for fear of giving rise to moral hazard is untenable. In a limited number of cases, state injections of capital will result in the government taking an ownership position in a SIB, which may be necessary to restore market confidence.

Bail-in tools can provide additional capital to strengthen bank balance sheets by converting creditor claims to equity when there is no danger of contagion, especially when the key cause of bank failure is idiosyncratic, for example, fraud. The use of bail-inable instruments needs to be balanced against the overall stress placed on the asset side of a bank’s balance sheet. An inundation of NPLs can negate the role of bail-inable instruments and capital buffers in maintaining banking system viability and stability.

To manage disproportionate stress placed on the asset side of a bank’s balance sheet, it is advisable that regulators adopt a broad definition of NPLs/NPEs to capture the widest range of distressed assets present in their financial system. Accounting treatments should avoid fair value and incurred loss accounting which underestimate banking system vulnerability. ECL accounting treatments provide a more accurate financial position in stable markets. However, the use of ECL accounting should be suspended when a sudden economic shock, such as the COVID-19 shutdown, results in a large concentration of NPLs. Delaying and spreading ECL reporting requirements over a longer time horizon will mitigate incidents of procyclicality, which can place subsequent pressure on bank capital buffers.

A key problem for AMCs is asset valuation. From an accounting perspective, bad debts are considered uncollectable. Thus, the chances of AMC profitability are low unless bad debts are bought at a steep discount that exceeds funding costs. Large exposures to NPL-linked financial instruments can complicate the design of AMCs to sequester distressed assets from banks. In these circumstances, retaining distressed assets on-balance sheet supported by government guarantees is the preferred option. Government guarantees that retain distressed assets on-balance sheet can lead to lack of control over the timing of sales, exposing governments to substantive risk and extensive capital injections. Guarantees should only be used when banks can be returned to viability and NPL sales can be controlled.

Debt restructuring requires legislative frameworks and infrastructure. If NPL legislation or infrastructure is absent or deficient, a programme should be designed that is expeditious and ideally takes an ex ante approach. Effective and expeditious balance sheet restructurings depend on legislation that builds suitable bankruptcy, arbitration and civil procedures. These requirements should not depress NPL sales and values or impede the emergence of distressed asset markets.

To incentivise NPL transfers, governments can guarantee NPL sales to private AMCs and AMC bond issues. NPL transfer efficiency is heightened by a market-based system because government guarantees require calibration to balance the competing incentives of transferring NPLs off-balance sheet with minimising AMC losses from NPL purchases. As guarantees expose taxpayers to risk and increase the cost of a programme, fees can be charged to offset costs and calibrated to mitigate abuse of the programme.

An AMC must be capable of maximising discretionary NPL sales. Ideally, NPLs are sold when market conditions yield profit and an efficient transfer. Successful NPL sales require a developed distressed asset market. In turn, successful distressed asset markets require expeditious legal processes. The optimum market-based restructuring solution for NPLs utilises private-sector AMCs, a tax regime that promotes distressed asset markets and a legal system that ensures the efficient and effective sale and transfer of NPLs.

Assuming these conditions are fulfilled, AMCs can be a very effective asset-side approach in NPL accumulations on bank balance sheets, strengthening capital ratios in the long term and enhancing banks’ capacity to restart lending. Focusing on asset-side balance sheet strengthening and adopting a pragmatic rather than dogmatic approach to bank crisis resolution will be of paramount importance for the support of robust economic recovery in the wake of the combined shocks of COVID-19 and the invasion of Ukraine for both developed and developing countries.

## Footnotes

1 Geanakoplos (2010).

2 Avgouleas (2015).

3 Brunnermeier et al. (2009), p 5 et seq.

4 This paper uses NPL ratios primarily sourced from the World Bank.

5 Minsky (1992, 1970).

6 Kiyotaki and Moore (1997). Naturally, causality is reciprocal.

7 Claessens (2014).

8 In the simplest terms: ‘Net present value is the present value of the cash flows at the required rate of return of your project compared to your initial investment, or ROI [return on investment], for a project or expenditure.’ See Gallo (2014). $\mathrm{NPV}={\sum }_{t=1}^{\mathrm{N}}\frac{{\mathit{Cas}h \mathit{flow}}_{t}}{{(1+i)}^{t}} - initial \, investment.$ Where ‘N’ is the total number of time periods for the cash flow being discounted; ‘*t*’ is the duration of the cash flow period; and ‘*i*’ is the discount or interest rate.

9 See Avgouleas (2020), ch. 8.

10 Georgieva (2020). For an updated account see Gopinath (2020).

11 Adler and Boissay (2020).

12 The most recent update of the IMF Global Financial Stability Report predicts a wave of bankruptcies that will put pressure on banks, see IMF (2020a), p 6.

13 See Arner (2007); Weber et al. (2014).

14 See Arner and Norton (2009).

15 Bholat et al. (2016), pp 22–23.

16 BCBS (2017), p 1; BCBS (2016), p 7.

17 World Bank (2002), p 3.

18 BCBS (2017), pp 1 and 8.

19 The term ‘non-performing loans’ is not uniform among jurisdictions. This paper adopts the IMF definition from Bloem and Freeman (2005), p 8.

20 Bholat et al. (2016), pp 36-37.

21 IMF (2015b), p 52.

22 BIS (2017), pp 1 and 6–7.

23 Bholat et al. (2016), p 21.

24 In the EU, the European Banking Authority issued guidelines in April 2020, see EBA (2020).

25 On the mechanics and effects of the Spanish dynamic pre-provisioning system adopted in the mid-2000s as a macroprudential measure, see Jiménez et al. (2012).

26 See Jiménez et al. (2014).

27 Klein (2013).

28 See Weber et al. (2016), pp 9–11.

29 Ibid., p 9. In particular, the authors of the paper note: ‘The prolonged recession led to higher default risk for large corporates and banks, which are typically low-default portfolios.’

30 The literature on financial dependence and growth is well established, see Rajan and Zingales (1998); Kashyap et al. (1994). Several recent studies have looked specifically at the feedback effects from NPLs to macroeconomic performance and have reached similar conclusions, e.g., Klein (2013); Nkusu (2011); Prasad and Espinoza (2010)*;* Bergthaler et al. (2015).

31 Aiyar et al. (2015a).

32 Aiyar et al. (2015b), Figure 2.

33 European Stability Mechanism (2016), pp 42–43.

34 World Bank Group et al. (2012).

35 The IMF employs a ‘nonperforming loans net of provisions to capital’ ratio as an indication of the extent to which losses can be absorbed before the sector becomes technically insolvent, see IMF (2015a) referring to IMF (2006), Part II, [6.15].

36 European Stability Mechanism (2016), p 4.

37 In fact, if a separate set of variables to what the European Banking Authority uses for its stress tests is employed, the impression of vulnerability is even stronger, see Acharya et al. (2016).

38 Ibid. Indicatively, the authors note that ‘[s]ince the start of the Banking Union in November 2014, European banks lost nearly half their market capitalization.’

39 For example, 80% of NPLs in Italy are loans to corporates, see Jassaud and Kang (2015), p 6.

40 Ibid., p 17; Aiyar et al. (2015b).

41 IMF (2015b), p 52.

42 See Jassaud and Kang (2015).

43 Avgouleas and Goodhart (2015).

44 See Avgouleas and Goodhart (2016).

45 Garrido (2016), p 6.

46 Title II of the Dodd–Frank Wall Street Reform and Consumer Protection Act of 2010 (Act (Pub L 111–203, HR 4173).

47 Directive 2014/59/EU establishing a framework for the recovery and resolution of credit institutions and investment firms, OJ L 2014 173/190, or BRRD.

48 Santos (2014), Ueda and Weder Di Mauro (2011), Li et al. (2011) and Morgan and Stiroh (2005).

49 Alfonso et al. (2014), Marques et al. (2013) and Gadanetz et al. (2012)

50 See Avgouleas and Goodhart (2016) and Avgouleas and Goodhart (2015).

51 Yet bailout costs may not be accurately measured unless the cost of the alternative—instability—is also considered, see Dewatripont (2014). With the US Troubled Asset Relief Program, public intervention may be recovered in the long term, which makes calculating the cost of public bailouts even more complex.

52 See Avgouleas and Goodhart (2019), ch. 2.

53 Haldane (2010); see also Haldane and Madouros (2012).

54 BCBS (2013), p 3.

55 FSB (2015b), p 5.

56 On the latter, see Avgouleas and Goodhart (2015).

57 FSB (2015b), p 10.

58 BCBS (2011), pp 62–63.

59 FSB (2016a), pp 9–18.

60 Avgouleas and Goodhart (2016).

61 On the distinction between applying bail-in to a bank that has failed for idiosyncratic reasons and a bank resolved due to systemic upheaval, see Avgouleas and Goodhart (2015); Avgouleas and Goodhart (2019), ch. 2.

62 Preconditions for an effective resolution regime include: (i) an established framework for financial stability, surveillance and policy formulation; (ii) an effective system of supervision, regulation and oversight of banks; (iii) effective protection schemes for depositors and other protected clients or customers, and clear rules on the treatment of client assets; (iv) a robust accounting, auditing and disclosure regime; and (v) a developed legal framework and judicial system. See FSB (2016b), p 13.

63 These cover: (i) statutory approaches; (ii) contractual recognition; (iii) temporary stays and early termination rights; and (iv) a bail-in tool. Contractual recognition supports cross-border resolution enforceability, for example, the write-down, cancellation or conversion of debt instruments. Where bail-in instruments are governed by foreign law, bail-in recognition clauses are to support debt instruments for home resolutions. See FSB (2015a), pp 7–8.

64 FSB (2011), p 3.

65 Ibid., pp 7–8.

66 Ibid., p 9.

67 Ibid.

68 Lindgren et al. (1999), p 65.

69 Enoch et al. (2001), pp 77–78.

70 Ibid., p 29.

71 Ibid., p 107. Authors’ calculations.

72 Ibid., p 39. Authors’ calculations.

73 Berg (1999), p 53.

74 Bank of Thailand (2001), pp 5 and 17.

75 Bank of Thailand (undated).

76 Bank Negara Malaysia (1999), ch. 4, p 12.

77 Bank Negara Malaysia (2001), ch. 4, p 14; Bank Negara Malaysia (2003), ch. 4, p 116.

78 Bank Negara Malaysia (1999), ch. 4, p 12.

79 Bank of Korea (1998), pp 17, 27 and 29.

80 Bank of Korea (1998), pp 38–39.

81 Kihwan (2006), pp 14–15.

82 Bank of Korea (1998), p 38.

83 Bank of Korea (2003), p 58.

84 Bank of Korea (2002), pp 49 and 51. Authors’ calculations.

85 World Bank (undated).

86 BIS (1999), p 91.

87 Ibid., p 93.

88 Ibid., pp 93–96.

89 See Hsu et al. (2007).

90 Ma and Fung (2002), pp 4 and 11–12.

91 World Bank (undated).

92 PwC (2020), p 3.

93 In April 2021, a state-owned AMC—Huarong—required support measures, see Caixin (2021).

94 Mitchell and Yang (2019).

95 Trivedi (2020).

96 Bloomberg (2020).

97 IMF (2020b), p 3.

98 Xiaomeng and Xiao (2019).

99 On TARP, see Board of Governors of the Federal Reserve System (undated).

100 SNB (2013), p 1.

101 StabFund or stabilization fund.

102 European Commission (2009), p 7.

103 UKFI (2010), p 4.

104 Asset Protection Agency (2010), p 20.

105 Board of Governors of the Federal Reserve: Board of Governors of the Federal Reserve System

106 See Avgouleas and Arner (2017).

107 ECB (undated).

108 European Commission (2014), pp 1 and 3.

109 See Avgouleas and Goodhart (2015), pp 3–27

110 European Commission (2014), p 5

111 BRRD, Chapter IV, Arts. 2-5.

112 ECB (2016), p 120.

113 BRRD, Art. 37(10(a)) and Recs. 73 and 75. For the advantages and disadvantages of this approach, see Avgouleas and Goodhart (2014).

114 Council of the European Union (2017a), [8].

115 Small and medium enterprises represent 16.7% compared with 7.5% for large companies and 4.7% for households, see Council of the European Union (2017b), [13] and [21].

116 European Council (2017).

117 European Commission (2018c).

118 Regulation (EU) 2019/876 of the European Parliament and of the Council of 20 May 2019 amending Regulation (EU) No 575/2013 as regards the leverage ratio, the net stable funding ratio, requirements for own funds and eligible liabilities, counterparty credit risk, market risk, exposures to central counterparties, exposures to collective investment undertakings, large exposures, reporting and disclosure requirements, and Regulation (EU) No 648/2012 (CRR II); and Directive (EU) 2019/878 of the European Parliament and of the Council of 20 May 2019 amending Directive 2013/36/EU as regards exempted entities, financial holding companies, mixed financial holding companies, remuneration, supervisory measures and powers and capital conservation measures (Capital Requirements Directive).

119 Regulation (EU) 2019/630 of the European Parliament and of the Council of 17 April 2019 amending Regulation (EU) No 575/2013 as regards minimum loss coverage for non-performing exposures.

120 European Commission (2018b).

121 E.g., EBA (2018); EBA (undated).

122 ECB (2017), pp 12–13

123 Ibid.

124 For the most recent EU pronouncement of this policy, see European Council (2018).

125 By building up capital buffers ex ante, banks will reduce the provision of credit, thereby reducing credit growth in the event of a credit bubble. However, these measures will affect credit growth in other times, which will make prudential backstops a very blunt regulatory instrument.

126 See European Commission (2018a).

127 European Commission (2018d).

128 World Bank (undated).

129 FROB (2012), p 7.

130 Ibid.

131 European Commission (undated).

132 Banco de Espana (2012b), p 40.

133 Sareb (2013).

134 See generally Banco de Espana (2012a).

135 Sareb (2013), p 1. Sareb is a public limited company with a 15-year lifespan to liquidate assets.

136 IMF (2013), p 9.

137 Banco de Espana (2013), p 13.

138 Ibid., p 22, Table 2.1.

139 World Bank (undated).

140 See NAMA (2020).

141 Commission of Investigation into the Banking Sector in Ireland (2011), p ii.

142 Honohan (2010), p 22.

143 NAMA Act 2009, s 2(a)-(b).

144 IMF (2010).

145 Department of Finance (Ireland) (undated).

146 NAMA (2014), p 12.

147 IMF (2015b), p 52.

148 NAMA (undated).

149 World Bank (undated).

150 FitchRatings (2014), p 1.

151 Donnery et al. (2018), pp 59 and 68.

152 World Bank (undated).

153 Bank of Italy (2012), pp 143–144.

154 World Bank (undated).

155 Bank of Italy (2015a), p 118.

156 European Commission (2015), p 1.

157 Nuova Banca delle Marche; Nuova Banca dell’Etruria e del Lazio; Nuova Cassa di Risparmio di Chieti; Nuova Cassa di Risparmio di Ferrara.

158 Reuters (2017).

159 Ibid.; Bank of Italy (2017), p 33.

160 Dipartimento del Tesoro (2016); Bank of Italy (2016).

161 Veneto Banca and Banca Popolare di Vicenza lacked sufficient resources to cover future losses, see Visco (2017), pp 4–5. Some retail junior bondholders were compensated for losses.

162 Jassaud and Kang (2015), p 17.

163 World Bank (undated); CEIC (undated).

164 Jassaud and Kang (2015), p 18. E.g., UniCredit, Intesa, KKR and Alvarez & Marsal.

165 The Economist (2016).

166 Quarati (2016).

167 IMF (2019), p 17.

168 Hellenic Financial Stability Fund (undated).

169 Bank of Greece (2014), p viii.

170 EBA (2016), p 12; World Bank (undated).

171 KKR (2016).

172 Reiser (2017).

173 European Commission (2019).

174 European Commission (2020).

175 World Bank (undated).

176 OECD (2011), p 77, referring to IMF Financial Soundness Indicators.

177 Ibid.

178 World Bank (undated).

179 Banco de Espana (2012b), p 40.

180 European Commission (undated).

181 NAMA Act 2009, s 12(2)(a) and (d).

182 Jassaud and Kang (2015), p 18.

183 Ibid., p 17.

184 Bank of Italy (2016), pp 34-35.

185 Bank of Italy (2015a), p 118.

186 Aiyar et al. (2015a), p 14.

187 Altman (2013), p 17.

188 Jassaud and Kang (2015), p 19 (Figure 14) referring to PwC.

189 S&P Global (2020).

190 World Bank (2020), p 7.

191 Statista (undated); Bureau of Economic Analysis (undated).

192 OECD (2020).

193 International Monetary Fund (2021).
